# Large Variability of Iodine Content in Retail Cow’s Milk in the U.S.

**DOI:** 10.3390/nu12051246

**Published:** 2020-04-28

**Authors:** Janet M. Roseland, Katherine M. Phillips, Kristine Y. Patterson, Pamela R. Pehrsson, Rahul Bahadur, Abby G. Ershow, Meena Somanchi

**Affiliations:** 1 U.S. Department of Agriculture, Agricultural Research Service, Methods and Application of Food Composition Laboratory, Beltsville, MD 20705, USA; kris.patterson@usda.gov (K.Y.P.); pamela.pehrsson@usda.gov (P.R.P.); 2 Biochemistry Department, Virginia Tech, Blacksburg, VA 24061, USA; kmpvpi@vt.edu; 3 Department of Nutrition and Food Science, College of Agriculture and Natural Resources, University of Maryland, College Park, MD 20742, USA; Rahul.bahadur@usda.gov (R.B.); drminisoma@gmail.com (M.S.); 4 National Institutes of Health, Office of Dietary Supplements, Bethesda, MD 20892, USA; ershowa@od.nih.gov

**Keywords:** iodine, milk, food composition, iodophors, intake, variability

## Abstract

Iodine intake is of contemporary public health interest. The recommended daily iodine intake is 150 µg for most adults, and milk is an important source of iodine in the U.S. diet. Iodine concentration in cow’s milk is affected by diet and iodine supplementation levels, milking sanitation practices, and other factors. Current analytical iodine data in U.S. retail milk are crucial for evaluating population-wide health outcomes related to diet. Samples of whole (3.25% fat), 2%, 1%, and skim (0–0.5% fat) milk were procured from 24 supermarkets across the U.S. using a census-based statistical plan. Iodine was analyzed by inductively coupled plasma mass spectrometry, including certified reference materials and control samples to validate results. No difference in iodine content was found between milkfat levels (F_3,69_ 1.033, *p* = 0.4). Overall mean (SEM) was 85(5.5) µg/serving (240 mL). However, the 95% prediction interval of 39–185 µg/serving for individual samples indicated high variability among individual samples. Given the recommended 150 µg iodine per day for most adults along with the study mean, one milk serving can provide approximately 57% of daily intake. Researchers, health care professionals, and consumers should be aware of iodine variability in milk, while additional research is needed to investigate the impact of iodine variability factors.

## 1. Introduction

Iodine is an essential element that is critical for synthesis of thyroid hormone and for normal human development, growth and metabolism [1]. Sufficiency of dietary iodine is again a current public health question in the U.S. and elsewhere [2,3,4]. Since diet is the source of iodine, adequate intake is required with a current recommended daily intake of 150 µg for persons 14 and older, 220 µg during pregnancy, and 290 µg during lactation [1,5]. Conversely, excess iodine intake can have adverse health effects, including thyroid dysfunction (i.e., goiter, hyperthyroidism), cancers, and autoimmune thyroid disease [6,7]. The tolerable upper limit for adults is 1100 μg/day [5], which is more than seven times the recommended U.S. intake for most adults. 

Major sources of dietary iodine include dairy products, eggs, fish, seaweed, iodized salt, and dietary supplements [4,8]. The intake of iodized salt has declined due to dietary guidelines for reduced sodium intake [3,9], increased consumption of processed and restaurant foods generally not prepared with iodized salt [3,10,11], and the popularity of various non-iodized salts [12]. As a result, foods rather than iodized salt have become increasingly important sources of iodine. Detailed data on the iodine content of foods are lacking [4], however, and iodine is not a required nutrient for food labeling in the U.S. Until 2019, iodine was not reported in the U.S. Department of Agriculture (USDA) nutrient database (https://fdc.nal.usda.gov) [2,8,13], although national food composition databases of at least 24 other countries include iodine [14]. Scientific publications suggest wide variability in the iodine content of the foods that are prominent dietary iodine sources [2,4,7].

Milk can have a significant iodine concentration. Dairy products provide 49% of total estimated daily iodine intake from food in the U.S. [15], making milk one of the most common food sources of iodine [16,17]. The concentration of iodine in milk is affected by the iodine content of the cows’ diet and iodine supplementation levels [18], presence or absence of goitrogens [19], sanitation practices that may or may not use iodine in different forms, levels, and modes of application [20], seasons and other factors [21,22,23].

The goal of this study was to provide an estimate of the iodine content of the most commonly consumed retail milks in the U.S. for inclusion in the U.S. food composition database (USDA, 2019), for estimation of population intake, and for obtaining a preliminary estimate of the variability of iodine concentrations in the retail milk supply. 

## 2. Materials and Methods

### 2.1. Sampling Plan

**Figure 1:**
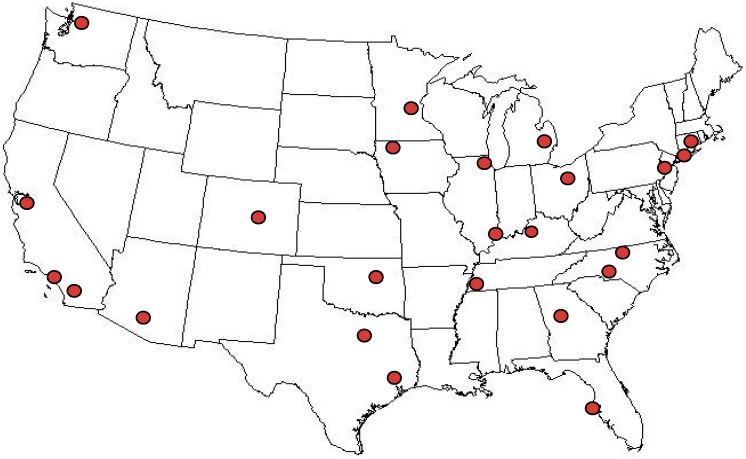
Sampling locations for retail milk. Locations were statistically selected as described in the Methods section.

The objective was to obtain a sample set that would provide nationally representative data for whole (3.25% fat), 2% fat, 1% fat, and skim (0–0.5% fat) fluid cow’s milk, the four most commonly consumed types of milk in the U.S. [24]. Milk was sampled from 24 locations (Figure 1) using the statistical nationwide sampling plan developed and implemented for the National Food and Nutrient Analysis Program (NFNAP) [25], as follows. The plan was a stratified design, based on population density data from the most current (2010) census data from the U.S. Bureau of the Census, and food sales data for retail outlets (ACNielsen, Inc., New York, NY, USA; proprietary). Counties and specific retail outlets for sampling were selected by “probability proportional to size” and sales volume, meaning that any county or store in the nation had a chance of being selected, but the higher the proportion of sales was to the total, the greater the probability of that county or store being selected [25]. Although a nationally representative sampling of retail milk, the sampling plan was not designed to measure the effect of all variables that could potentially affect the iodine content of milk such as agricultural practices in specific locations, seasons, brands, or processing plants. 

### 2.2. Sample Procurement

Samples were procured in April and May 2018. From each retail outlet, one half-gallon container of each of the four fat levels of milk was purchased on the same day. The brand occupying the largest display space (usually the store brand or major local/regional dairy brand) was procured. The samples were kept in original containers, packed in insulated coolers with ice packs to maintain refrigerated temperature, and shipped via overnight express delivery to the Food Analysis Laboratory Control Center (FALCC) (Virginia Tech, Blacksburg, VA, USA), according to established methods [26]. Milk containers were inspected upon receipt to ensure integrity of packaging and then kept refrigerated at 2–5 °C until preparation for analysis. 

### 2.3. Sample Preparation

Milk from each sample unit was dispensed into subsamples for analysis of iodine and other nutrients (which were also analyzed for entry into the U.S. food composition database) within six days of receipt and always before the labeled expiration date. Subsampling methods for nutrient analyses have been previously described [27]. Subsample containers were sealed under nitrogen and frozen (−60 °C) until analysis. 

Milk samples were batched with control materials including certified reference materials (described in Section 2.5) and shipped on dry ice via express overnight delivery to the analytical laboratory whose performance on iodine analysis had been previously validated [28]. 

### 2.4. Iodine Analysis

Iodine was analyzed by inductively coupled plasma mass spectrometry (ICP-MS) after digestion of the sample with potassium hydroxide, followed by stabilization with ammonium hydroxide and sodium thiosulfate (method 2012.15) [29]. The analytical sample weight for the milk was approximately 2 g.

### 2.5. Quality Control

Sample identities were blinded, with only the milkfat content noted (e.g., skim, 1%, 2%, whole). Each sample was given a batch identification in which it was grouped with a control sample(s) for analysis. Control samples included in-house control composites (CCs) (whole chocolate milk (“Chocolate Milk II CC”) and 2% milk (“2% Milk CC”)), developed for the National Food and Nutrient Analysis Program (NFNAP) using established methods [30,31], and commercially available standard reference material (SRM) with a certified value for iodine (National Institute of Standards and Technology (NIST) SRM®1849a Adult Infant Nutritional Formula) [32].

To document the accuracy and the precision of results, each assay batch of approximately fifteen milk samples included one of the in-house control materials, while one batch had an additional control which was SRM®1849a. The percent relative standard deviation (RSD) was calculated for the multiple values for each CC, and the ratio of assayed RSD to expected RSD (“HorRat”) was calculated as: RSD_assayed_/((Mean_assayed_/100/1,000,000)^^-.1505^), with the absolute value expected to be ≤2 [33].

Results for SRMs and CCs were evaluated against certified limits (for SRMs) and expected ranges for CCs. If a control sample value was outside the acceptable range, the batch of samples was re-analyzed. Each milk sample was analyzed in singlicate. A few values were unusually high, with one of the values twice as high as the average; this one sample was re-analyzed, giving a result that was approximately 60% of the initial value. Additional samples were then re-analyzed, including that outlier sample and several randomly selected samples. The repeat analyses were within 10% of the initial values, except for samples from the batch with the originally high sample. As a result, all samples from the questionable batch were re-analyzed; those values were used instead of the original values. 

### 2.6. Data Analysis

The goal of the study, reflected in the sampling plan, was to estimate the nationwide weighted average iodine content of U.S. retail milk (skim, 1%, 2%, and whole). Statistical analyses were done to determine the mean and 95% confidence interval (CI) for each fat level of milk, and to test for differences in the mean and variance among milkfat levels. All statistical analyses were performed with R software (R Core Team; Vienna, Austria [34]). The total of 96 observations resulted from analysis of four milkfat levels from each of the 24 retail locations sampled. For nutritional relevance, all analytical results in µg per 100g were transformed into units of µg per 240 mL, which is one serving [35]. Hereafter, the term “serving” refers to 240 mL (equivalent to 1 cup). 

Least square means, 95% confidence interval, and 95% prediction interval (range of values for predicting a new individual observation with a 95% probability) were calculated using a linear mixed-models approach (R package: lme4) [36] after log (base = 10), transforming the data. Log-transformation was deemed appropriate for the statistical analyses, since there was a positive skew in the data and generally an increase in variance in iodine values among the four milkfat levels, as the value of iodine increased. The results of the analysis were then back-transformed from log to the original scale. The best fitting model (based on AIC [37] and log-Likelihood [38] tests) had a fixed effect for the overall mean of iodine for milk in µg/240 mL and random effects for locations.

Tests for outliers and influential observations/groups were done after log-transformation of the data for purchase location and individual observation. For this, Cook’s distance was calculated [39] by leaving out one observation or a group of observations at a time. No observations or locations had a Cook’s distance greater than 0.5 and, hence, no observations were cause for concern.

For testing whether the four milkfat levels differed in iodine content, a mixed-effects model was built with fixed effects for the four types. One-way ANOVA and a pairwise comparison test were done (R package: emmean) [40], with the Tukey method used for adjusting p values for a family of four estimates. 

To test for differences in variance between milkfat levels, the best-fit model was updated to allow each level to have its own variance. This updated model was then compared with the best fit model based on log-Likelihood tests [38] to determine whether different variances for each level improved model fit.

## 3. Results

### 3.1. Sample Descriptive Information

The 96 samples (one sample of each milkfat level from each of 24 sampling locations) included 26 different brands, which were a mix of national company and store names, reflecting the entities that carried out the post-farm processing and packaging. Each processing plant that produced each carton of milk was determined using codes on the package labels [41], indicating that the same plant processed all four milk types sampled at a given retail location, except in one identifiable case (the four samples from the Pennsylvania store came from two different plants). One sample from one of the North Carolina stores had an unreadable code. For each sampling location, the processing plant was either in the same state or an adjoining state. Varying expiration dates on package labels of the four samples from each location also suggested that they were typically not all processed on the same day. 

### 3.2. Quality Control

Table 1 summarizes the results for the certified reference materials (RMs) and in-house control materials (CC) analyzed with the milk samples, along with measures of reproducibility and comparison with expected values. RM results were within the certified ranges and CC results were within previously established limits (Section 2.5). HorRats of <0.5 demonstrate acceptable reproducibility in all cases (Section 2.5). Importantly, the values for controls were obtained across multiple analytical batches, supporting a lack of analytical bias among milk samples assayed in different batches/days, and also providing an estimate of the precision of single measurements on individual samples. 

### 3.3. Iodine Content of Retail Milk 

**Figure 2:**
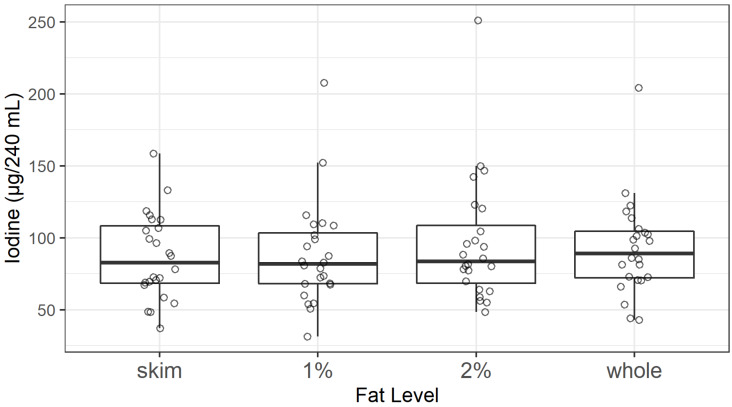
Iodine content of U.S. retail milk samples by fat level. Median of each milkfat type is depicted with heavy horizontal line, showing no statistically significant difference between milkfat levels.

The mean iodine content did not differ among milkfat levels (skim, 1%, 2%, and whole) (F_3,67.3_, *p* = 0.4) (Figure 2). Additionally, there were no statistically significant differences among the variances for the 24 samples of each of the four milkfat levels (α=0.05, data not shown). This finding is consistent with commercial milk production methods, in which cream is skimmed from large batches of milk and the final fat levels are achieved by adding some cream back into the milk to produce precise fat levels [42,43]. Therefore, all values (*n* = 96) were used to calculate the nationwide average and confidence interval for the iodine content of retail milk in the U.S. 

**Figure 3:**
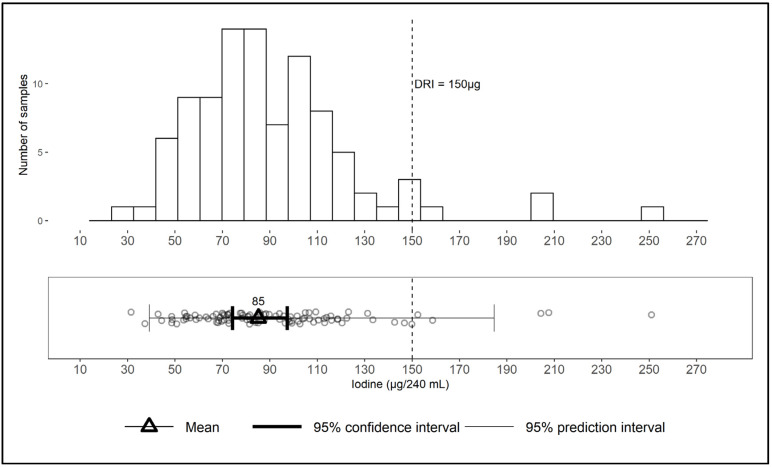
Iodine content of retail samples of milk in the U.S. (*n* = 96) per 240 mL serving, shown as distribution, mean, 95% confidence interval, and 95% prediction interval. DRI = Dietary Reference Intake [5].

The overall mean and standard error (SEM) across all four milk types is 85 (5.5) µg iodine per serving, with a 95% confidence interval of 74–97, median of 84, and range of 31–251 (Figure 3). The log-normal distribution of values, skewed towards the right (higher concentration), is shown in the upper panel of Figure 3. The prediction interval of 39–185 µg/serving is the range in which the iodine content of a new sample would be expected to fall with a 95% probability. The prediction interval for the iodine content of an individual sample (39–185) is much wider than the 95% confidence interval around the overall mean (74–97) (Figure 3), suggesting that, although the mean is a reliable estimate of the population average iodine content of milk, it poorly predicts the content of an individual sample. 

## 4. Discussion

### 4.1. Other Reports on Iodine in Milk

Numerous studies on the iodine content of milk have been published, but ours includes the most up-to-date nationwide statistically based U.S. sampling and analytical methods for iodine. The last analysis of iodine in nationally sampled U.S. retail milk was reported by the Food and Drug Administration (FDA) as part of the Total Diet Study (TDS) spanning the years 2003–2012, with sampling divided into four geographic regions [8,44]. In that analysis, whole milk had iodine concentrations (mean ± standard deviation) of 98.4 ± 19.2 and skim milk had 101 ± 28.8 µg/240 mL serving; *n* = 32 for each milkfat level. An earlier FDA TDS evaluated whole milk over time and location with samples obtained quarterly from four regions, from 1982 to 1990 [45]. In that study, iodine averaged 56 µg/serving; concentrations were highest in the winter and lowest in the summer and were higher in central and western regions than in the eastern region (*p* ≤ 0.05) [45]. The higher iodine levels in the period 2003–2012 compared to the period 1982–1990 were probably due to the impact of increased iodophor disinfectant use during milking and higher iodine content in the cow’s diet [8]. Caution should be used when considering data from various studies, due to differences in analytical methods among studies, such as availability or lack of published data for certified reference materials and analytical methodology. In fact, colorimetry was used in the 1982–1990 FDA study [45], as well as in the later FDA study [44]; however, ICP-MS is used for their present work. Additionally, the FDA studies lack published data for commercially available reference materials, so any potential laboratory or method bias is unknown. 

Besides nationwide U.S. samplings, other studies of iodine in milk have been reported. A city-wide retail milk sampling during the period 2001–2002 [46] reported an iodine content of 103.1–117.9 µg/240 mL (95% confidence interval; *n* = 36), which is higher than the mean iodine content in the present study (85 µg/240 mL). In contrast, an 11-state retail milk study reported a mean iodine content of 21.4 µg/240 mL, with a relatively wide range of values, from 2.3 to 91.7 (*n* = 39) [47]. 

As an example of iodine being studied and reported in other countries, in Denmark’s national database [48], iodine in whole milk is 10.7 µg/100 g and iodine in skim milk is 11.7 µg/100 g (equivalent to 26.7 and 28.1 µg/240 mL, respectively; *n* = 7). While the findings of individual studies are informative, especially with overlapping ranges in some cases despite differences in means, the results should not be compared directly. The studies vary in their sampling plans, analytical methods, geographic locations, and other factors. For example, the low Danish values could derive from markedly different international factors relating to cattle nutrition or analytical detection methodology. Another potential explanation of variability, if comparing retail sampling locations in different states, is that states have regulations pertaining to milk production such as feed, sanitation, and manufacturing standards, in addition to adherence to federal regulations [43,49].

Just as we found no significant difference in iodine content among retail milk with differing fat content, similar results were obtained in other studies [50,51,52]. However, one study found significantly higher iodine levels in skim than in whole or in semi skimmed [53], although the difference was inconsequential in terms of nutritional impact. 

### 4.2. Factors Influencing Iodine Levels in Milk

**Figure 4:**
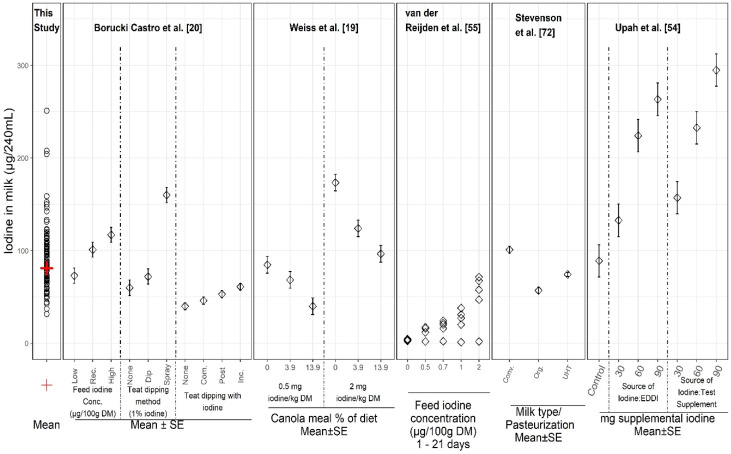
Iodine concentrations in individual milk samples from this study (left column) and from several reports on controlled experimental studies of variables affecting the iodine content of milk. Abbreviations: Rec = recommended; DM = dry mass; SE = standard error of mean; None = no iodine; Com. = complete; Inc. = incomplete (see [20] for details); Conv = conventional (not organic) milk, Org = organic milk; UHT = ultra-high temperature pasteurization; EDDI = Ethylenediamine dihydriodide (80% iodine), Test Supplement = proprietary mineral supplement (40% iodine) (see [54] for details).

Many factors are known to influence iodine concentrations in milk. Figure 4 illustrates results of select studies reporting controlled experiments of discrete variables on iodine in milk, along with results for individual samples from the present study. Variables include levels and sources of iodine supplements, dietary goitrogens, iodine preparations and treatments for sanitation during milking, and processing methods. In the controlled studies, milk iodine concentrations ranged from 2 to 299 µg/240 mL. The iodine concentration in the 96 samples from our current study fell within the range of the values from the studies in which iodine was supplemented and/or iodophors were used during milking, suggesting that the large sample-to-sample variation in iodine in our study (31–251 µg /240 mL) might be explained by a combination of these variables. Our sampling plan was not designed to measure or assess specific sources of variability but rather to be statistically representative of the U.S. retail supply and for estimating the population-wide average milk iodine content and variability. 

As illustrated in Figure 4, iodine supplementation has a large influence on the iodine content of milk. For example, increased iodine intake of dairy cattle resulted in increased milk iodine concentration [54,55]. In addition to different levels and forms of supplemental iodine, feed and plant materials vary in iodine content due to passive iodine uptake from water, soil, and air, or by contamination, and can affect iodine levels in milk [21,22]. For example, cows grazing on pasture and fodder from higher iodine soil have produced milk with greater iodine concentrations than cows grazing on lower iodine pasture [56]. Similarly, animals grazing in sea coastal environments, which are high-iodine areas [57], or consuming hay which has elevated iodine due to drying [58], could be expected to produce iodine-enriched milk [56]. Conversely, the correlation between soil iodine and plant iodine frequently is poor, since plant species very greatly in their absorption and retention of iodine from the soil [59]. Nonetheless, our study was not designed to compare results to specific regions for determining whether iodine concentrations in milk correlated with the lowest iodine areas of the U.S. (Great Lakes, Appalachian, and Northwestern regions, a geographic area known as the “goiter belt”) [60]. 

Although iodine supplementation provides health benefits to cattle, the amount fed must be limited to avoid excessive iodine concentrations in milk and dairy products [19]. Therefore, supplemental iodine intake in dairy cows is regulated in the U.S. [11,61,62]. The maximum recommended iodine concentration in milk is approximately 500 μg/L (120 μg/240 mL serving), but there is no regulatory limit [19,63]. 

Season has been shown to influence dairy cattle’s intake of iodine. Iodine concentrations were generally found to be greater in winter (16–128 µg/240 mL) than in summer (8–104 µg/240 mL) across numerous studies in various countries [23]. A study in the U.S. found significantly higher mean iodine in retail milk in winter (116 ± 23.1 µg/250 mL) than in summer (91.3 ± 16.6 µg/250 mL) [46]. Significantly higher iodine levels in winter were also observed in Norway, with higher results in winter presumably due to more supplemented diets enriched with iodine compared to summer diets [64]. Seasonal changes in types of feed, influenced by availability or price, are additional explanations for seasonal iodine variations in milk [23]. In the present study, all samples were from one sampling point (April–May). Nonetheless, differences by season cannot be generalized without accounting for attributes of different dairy production systems. Some systems do not include grazing or outdoor exposure and thus would likely include year-round micronutrient supplementation [52]; these would not be expected to result in a measurable seasonal effect.

Iodine in milk can vary considerably due to other influential factors, such as milking management practices and the presence of goitrogens (GSL) in the cows’ diet [22,65]. For example, canola and rapeseed meals in feed reduce milk iodine concentrations by inhibiting mammary gland iodine uptake [19]. Conversely, iodine concentration in milk can be elevated by use of iodine-based teat dip before and after milking to control mastitis [20,22]. Considerable variation in milk iodine content has been seen in investigations of teat disinfection practices, due to differences such as iodine strength, formulation, and teat dipping versus spraying [20,66]. Iodophor teat dips were reportedly used by 53% of U.S. dairy operations for premilking and by 67% of operations for post-milking, indicating the prevalence of iodophor use [67]. Although the amount of iodine added to supplement cattle feed is limited by the USDA, using iodophors as disinfectants is allowed [49,67,68] and iodine levels in U.S. milk can vary substantially [20]. Processing plants are not a likely source of iodine in milk, however, since less than 5% of them are estimated to use iodophors for sanitizing; instead, most plants use peracetic acid and fatty acid-based sanitizers [68,69].

Other causes of iodine variability in milk have been hypothesized. For example, iodine loss during pasteurization has been observed in some studies [22,70,71] but not in others [52,55]. Also, ultra-high-temperature (UHT) processed milk had 27% lower iodine than conventional retail milk and organic milk had 44% lower iodine than conventional milk in a retail study across the United Kingdom (Figure 4) [72]. Iodine concentration increased from summer to winter for all three milk types. The results were in agreement with an earlier study [51]. Iodine differences seemed to coincide with the differing amount of iodine in the cows’ diets according to the season and due to the unique characteristics of the organic diet. Reasons are largely unknown for lower iodine in UHT milk versus conventional milk [72]. Findings suggest that consuming organic or UHT milk instead of conventional milk may increase the risk of suboptimal iodine intake [51]. 

### 4.3. Importance of Quality Control and Reference Materials

An important aspect of our study is the analysis and reporting of results for a commercially available RM and in-house control materials in every analytical batch of samples. These data are critical to ensure consistent assay performance across samples analyzed in different runs. Results for RMs with certified iodine content are critical to validate the accuracy of results, and data for commercially available materials allow data from different studies to be compared, by enabling the distinction of analytical variability from actual sample or treatment differences (as discussed previously [30,73]). Maintaining continuity of control samples that are used in the analyses of food samples across time and across studies has been an integral part of the NFNAP [28,30] for analytical data in the USDA’s food composition database. These types of data are essential to interpreting literature data from various studies among different laboratories, methods, samples, and across time, and should be reported by all researchers publishing food composition data. The difficulties in iodine analysis are described by Todorov and Gray [74] including information on the lack of specificity and less than desirable detection limits for many methods. Using ICP-MS to analyze iodine provides a method that is both selective and sensitive [74], and our study has demonstrated that quality control samples in each analytical batch, as well as replicate analyses, are critical to assure the accuracy of results and to document the contribution of analytical uncertainty. 

### 4.4. Implications for Nutrition Research and Use of Food Composition Data

In terms of milk intake, daily fluid milk consumption per person in the U.S. ranges from approximately ¾ to 1¾ servings (240 mL= one serving) on average among different age groups of 2 years and older, and is highest in males aged 2–5 years [75]. The Dietary Guidelines for Americans (DGA) recommend three servings of fat-free and low-fat dairy foods daily for people of ages 9 and older, based primarily on these products’ contribution of calcium, phosphorus, vitamin D (when fortified), vitamin A, riboflavin, vitamin B12, protein, magnesium, potassium, zinc, choline, and selenium [76]. The healthful benefits of these nutrients and others in fluid milk, such as iodine, are widely recognized [77].

From a statistical perspective, the results of the present study indicate that there is a 95% chance that the iodine mean intake for the population lies between 74 and 97 µg/serving of milk, which can provide a useful range for estimating population-based intake. Simply using a mean value might not be accurate for different points in time of collection or collections of samples at different locations with different milk sources. In other words, the mean does not take into account the uncertainty in estimating the mean iodine content in milk, although the 95% confidence interval does. Based on the confidence interval, there is a 95% chance that the population mean for iodine intake per serving has a value that contributes between 50% and 65% of the DRI. Users of food composition data should consider these distinctions in order to avoid applying a population-wide mean value inappropriately. 

When applying this study’s summary statistics, it is important to realize that the statistic used can dramatically affect the predicted dietary iodine shortfall or excess amount, as demonstrated by Carriquiry and colleagues [78]. Care should be taken to choose the appropriate statistic for the context. For example, if a single point estimate is to be used for public health estimates, then based on our results, the U.S. population on average receives 85 µg iodine from a serving of milk, which is approximately 57% of the Dietary Reference Intake (DRI) daily recommendation of 150 µg iodine for most people ages 14 and older [5]. On the other hand, estimated iodine intake from milk for an individual could be quite different. The 95% prediction interval shows the range of values to expect for a new observation. That is, the predicted iodine content of a new sample of U.S. retail milk has a 95% chance of being between 39 and 185 µg iodine/serving. These calculations imply that an individual in the U.S. could get as little as 26% or as much as 123% of the iodine DRI from one serving of milk depending on where the milk was purchased. However, this interval provides an estimate for one individual sample at one time, rather than for the iodine intake of the population. Furthermore, excess intakes of iodine which exceed the daily recommended intake for adults of 150 µg may impact iodine status in some vulnerable individuals [79]. This could include older people, individuals with chronic thyroid disease, and fetuses and newborns.

Thus, the authors caution that although some data users may choose to use 85 µg/serving as the national average, the variability expressed as the confidence interval around the mean as well as the prediction intervals should also be acknowledged and considered. It is necessary to include variability in intake calculations, especially for nutrients which have high variability and wide range in concentration. Thus, the authors caution against using database mean values alone when calculating intake for nutrients for specific populations. The broad range of prediction values observed in this study illustrates the need to examine iodine content and variability over a larger span of time at various retail locations, in order to confirm that iodine levels are appropriate. 

These new data have been incorporated into FoodData Central (FDC), the USDA’s new food composition database (USDA, 2019) [80]. The unique strength of FDC is the inclusion of individual sample values and their characteristics, which can connect food composition with agricultural practices and health outcomes using ontological information. Milk iodine data were needed for FDC for several reasons: a) to assist in assessing current U.S. dietary iodine intake; b) to determine levels, which were previously unavailable; c) to obtain estimates of variability; d) to identify issues which could result in unexpected values in samples or which could show the need for additional research. Mean, median, and range per milkfat type are provided in FDC and, in this report, the statistics are presented across types, since no statistical difference was found between types. These data were not available in the USDA database until the recent release of FDC. 

While these results apply to milk samples representing the U.S. food supply, implications for international data are appropriate. In order to comprehend and evaluate the state of global iodine nutrition status, we must be able to characterize the iodine content of national food supplies and imported/exported foods. This is critical for estimation of individual iodine status, nutrition research, treatment of thyroid diseases, and public health guidance. For example, in New Zealand, to compensate for low iodine in the food supply, bread is fortified with iodine [81]. Though foods like milk contribute significantly to iodine intake, in countries where iodine-containing dietary supplements are regularly consumed, these too must be characterized by iodine content. Total iodine intake and variability of iodine across geographic areas, agricultural practices, and processing practices should be an essential part of surveys if the relationship between intake and thyroid function within and across populations is to be understood. 

### 4.5. Limitations

This study provides a snapshot of milk iodine content and variability. As previously stated, the goal was to estimate iodine content in four milkfat levels from retail sampling locations, and thus could not measure the extent of specific sources of variability or the effect of each specific factor. A larger sample set obtained in various seasons and multiple retail sampling locations would narrow the confidence interval and prediction interval and help resolve questions as to the degree to which iodine varies over time and place, and the effects of seasonal differences or other factors. The results illustrate the value of investigating the impact of processing plants over time on iodine variability. Even within a processing plant, milk will likely be a mixture coming from multiple farms [43,82]. Each farm has varying feed regimens and milking sanitation practices. Furthermore, higher iodine concentrations from one farm will be diluted by milk of lower concentrations from other farms [45]. 

Variance between milkfat levels could not be determined in this study because samples of different milkfat levels within the same processing plant came from different batches. Thus, a larger sample size with more plants, brands, and batches could enable hypotheses testing on the impact of these factors. A next step of investigation would be a prospectively designed study to characterize the extent of iodine variability over time and multiple retail locations. It would be informed by findings from this study, scientific literature on factors affecting iodine in milk, and knowledge of specific milk production and processing practices. The ultimate aim would be to enable the modelling of iodine intake based on known values instead of reliance on database values alone. 

## 5. Conclusions

Milk contributes meaningfully to the DRI for iodine on a population-wide basis in the U.S., but actual intake in individuals cannot be accurately estimated using nationwide mean iodine concentration, due to large sample-to-sample variability and lack of information on the consistency of differences among samples and sampling locations. If, theoretically, there were a nationwide or localized reduction in use of iodine for sanitation during milking, a noticeable reduction in iodine intake from milk could result, since this is a major contributor to the iodine content of milk, given physiological iodine limits in cows influenced by their nutrient requirements [18,83]. Nutrition researchers should be aware of iodine variability in milk and the impact of changes in sanitation practices, especially for individuals closely monitoring iodine intake. Variability also should be considered when estimating population iodine intake. 

Final analytical nutrient data and descriptive information per sample unit for four types of retail milk were added to the USDA database, providing current estimates of the mean iodine content and variability, which were not previously available. These data can serve as the foundation for future studies to help keep pace with changes over time in agricultural and manufacturing practices by reflecting the content of retail milk products. Since iodine status is a public health concern, further research is needed to understand factors influencing iodine content and variability of milk and the degree of consistency of these factors within the food supply. 

## Figures and Tables

**Table 1:** Results for control samples analyzed with milk samples.

Control Material	*n*	Mean Iodine (µg/ 100 g)	SD	% RSD	HorRat ^a^	Minimum	Maximum	Previous Mean (Range, *n*) ^b^	Expected ^c^
Chocolate Milk (Control Composite II) ^d,e^	6	41.9	1.03	2.5	0.3	40.6	43.1	40.9 (1)	*n*/a
2% Milk (Control Composite) ^d^	3	32.2	0.31	0.9	0.1	31.9	32.5	*n*/a	*n*/a
NIST SRM®1849a Infant/Adult Nutritional Formula ^f^	1	133						127 (118–134, 7)	118–140

SD = standard deviation. RSD = percent relative standard deviation. ^a^ Assayed RSD/expected RSD, calculated according to Horwitz and Albert [33], and described in Section 2.5. ^b^ For samples of material assayed with other foods for the National Food and Nutrient Analysis Program [30,31]. ^c^ Certified mean ± uncertainty [32]. ^d^ CC=control composite developed for the National Food and Nutrient Analysis Program [30]. ^e^ Whole chocolate milk. ^f^ NIST SRM=National Institute for Standards and Technology Standard Reference Material (Gaithersburg, MD, USA) [32].
